# Long-term neurodevelopment in children with resected congenital lung abnormalities

**DOI:** 10.1007/s00431-023-05054-5

**Published:** 2023-06-16

**Authors:** Louis W. J. Dossche, Casper M. Kersten, Tabitha Zanen – van den Adel, René M. H. Wijnen, Saskia J. Gischler, Hanneke IJsselstijn, Andre B. Rietman, J. M. Schnater

**Affiliations:** 1 Department of Pediatric Surgery, Erasmus MC Sophia Children’s Hospital, Rotterdam, the Netherlands; 2 Department of Orthopedics, Section of Physical Therapy, Erasmus MC Sophia Children’s Hospital, Rotterdam, the Netherlands; 3 Department of Child and Adolescent Psychiatry/Psychology, Erasmus MC University Medical Center, 3015 CN Rotterdam, the Netherlands

**Keywords:** Congenital lung disease, Lung malformation, Neurodevelopment, Long-term outcome, Motor development, Neurocognitive development

## Abstract

To determine whether children who underwent resection of a congenital lung abnormality (CLA) are at higher risk for neurodevelopmental impairments than peers in the general population. The study population consisted of children born between 1999–2018 who underwent resection of a symptomatic CLA. Neurocognitive development (intelligence, memory, attention, visuospatial processing, executive functioning) and motor function of this population are monitored through our structured, prospective longitudinal follow-up program at the ages of 30 months, 5, 8, and 12 years. We compared study population scores with Dutch norm values using one-sample t-tests and one-sample binominal proportion tests. Forty-seven children were analyzed. The 8-year-olds showed significant impairments in sustained attention through the Dot Cancellation Test (mean z-scores -2.4; [-4.1; -0.8], p = 0.006 and -7.1; [-12.8; -1.4], p = 0.02 for execution speed and fluctuations respectively). Visuospatial memory was impaired at 8 years, though only in 1 out of 3 assessment tools (Rey Complex Figure Test z-scores (-1.0; [-1.5; -0.5], p < 0.001). Further neurocognitive outcomes were unimpaired at all tested ages. Regarding motor function outcomes, mean z-scores of total motor functioning were unimpaired across assessed ages. However, at 8 years, significantly more children than expected had definite motor problems (18% vs 5%, 95% CI [0.052; 0.403], p = 0.022).

*Conclusion*: This evaluation reveals impairment in some subtests of sustained attention, visuospatial memory and motor development. However, globally, normal neurodevelopmental outcomes were found throughout childhood. We recommend testing for neurodevelopmental impairments in children who underwent surgery for CLA only if associated morbidities are present or if caregivers express doubts about their daily functioning.

**Table:** 

**What is Known:** *• In general, surgically managed CLA cases seldom suffer from long-term surgery-related morbidity and show favorable lung function.*	
**What is New:** *• Long-term neurocognitive and motor function outcome appear unimpaired within surgically managed CLA cases. We recommend testing for neurodevelopmental impairments in children who underwent surgery for CLA only if associated morbidities are present or if caregivers express doubts about their daily functioning.*

**Supplementary Information:**

The online version contains supplementary material available at 10.1007/s00431-023-05054-5.

## Introduction

Congenital lung abnormalities (CLA) comprise numerous anatomical anomalies of the respiratory system: congenital pulmonary airway malformation (CPAM), bronchopulmonary sequestration (BPS), bronchogenic cyst (BC), congenital lobar overinflation (CLO), bronchial atresia, and ‘hybrid’ lesions [1]. The current incidence of CLA is 4/10,000 live births, while the number of prenatally diagnosed cases is rising – probably due to the implementation of prenatal ultrasound as standard-of-care and the improved resolution of these images [2, 3]. The gold standard for confirming the diagnosis of CLA is a postnatal chest CT during the first year of life [4]. It is estimated that fewer than 5% of children develop symptoms in the first five years of life, but strong evidence is lacking [5, 6]. From 36–97% of children born without symptoms remain asymptomatic throughout childhood [7–10]. In fetal life, CLA often regress spontaneously and become undetectable during repeated prenatal ultrasound assessment. Still, a chest CT scan after birth detects almost all lesions (98%) [11].

Clinical manifestations of CLA include respiratory distress, cardiovascular overload, mediastinal shift, pneumothorax, and recurrent lower respiratory tract infections [12–16]. Furthermore, CPAM lesions harboring KRAS or other oncogenic driver mutations, may have an increased risk for malignant degeneration [17]. In general, symptomatic CLA lesions are surgically resected but consensus on the management of asymptomatic lesions is lacking. Currently, there is no evidence on the optimal management and associated long-term outcomes of the different approaches to management [18–20]. Some advocate the resection of all CLA in the first year of life to prevent the onset of clinical manifestations [21]. Others practice a conservative management of asymptomatic CLA with routine follow-up [5], which would evade the general anesthesia and surgery that are thought to negatively influence long-term neurodevelopment [22, 23]. Multiple studies in recent years have addressed the optimal management of asymptomatic CLA, mainly focusing on CLA-related morbidity and surgery-related complications [24]. Several studies have investigated the post-operative lung function and exercise capacity in surgically operated CLA-patients, showing heterogeneous results [25–28]. Insights on the long-term effects of CLA-related surgery on neurocognitive and motor development are lacking.

Gaining insight on the neurodevelopment of children with resected CLA could improve the counselling of parents whose child is a candidate for CLA-related surgery and help manage future perspectives on their child’s development throughout childhood. We hypothesized that surgery for symptomatic CLA may put children at risk for neurocognitive and motor impairments, as exposure to general anesthesia in childhood and cardiac surgery in infancy have been associated with inferior neurodevelopmental outcomes [29]. Furthermore, survivors of neonatal critical illness and patients with congenital diaphragmatic hernia are more likely to experience motor function problems in childhood [30].

Hence, we longitudinally evaluated neurocognitive and motor performance in children that underwent resection of CLA, and compared their results to those of healthy, age-matched peers through standardized norm values.

## Methods

### Population

**Fig. 1:**
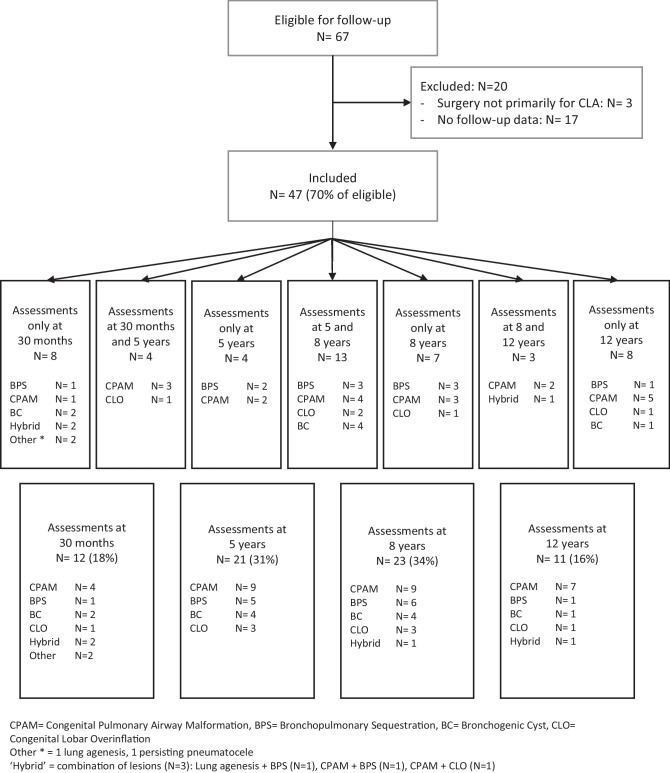
Flowchart of patient inclusion

All children born between January 1999 and July 2018 with chest CT-confirmed CLA who underwent surgery at our tertiary university hospital were eligible for inclusion in this chart review. The policy in our hospital towards patients with asymptomatic CLA is conservative management with routine follow-up. Consequently, this evaluation of neurodevelopment covers children with CLA-related clinical manifestations. In our center, all children that undergo CLA related surgery were prospectively enrolled in a structured surgical longitudinal follow-up program that was offered as part of the standard of care until the age of 17 years, followed by transfer to a specialized pulmonologist for adult care [31]. During regular post-operative follow-up visits, scheduled at the ages of 30 months (2.5 years), 5, 8, and 12 years, these children underwent several standardized developmental assessments (Fig. 1). In 2014, the assessment batteries used in the follow-up program were updated. As results from the updated tests are not interchangeable with their precursors, we used data collected between January 2014 and January 2021.

The Medical Ethical Review Board of the Erasmus University Medical Center approved this retrospective study design and waived the need for informed consent (MEC-2021-0185). All parents and children were informed that outcome data were used for research purposes.

### Patient characteristics

The following perinatal, clinical, and CLA-related characteristics were retrieved from the electronic patient files: sex, gestational age, prematurity – defined as gestational age at birth < 37 weeks –, birth weight, low birth weight – defined as birth weight < P10 for gestation –, type of CLA, presence of associated morbidities, age at onset of symptoms, age at surgery, and surgical approach.

### Neurodevelopmental outcomes

We used validated and standardized Dutch versions of the below mentioned neurodevelopmental tests with corresponding norm values whenever these were available.

### Neurocognitive assessments

Validated neurocognitive tests were administered to assess skills in five domains. Detailed information on these tests is shown in Supplemental file 1. All neurocognitive assessments were carried out by certified psychologists.

#### Intelligence

Intelligence was assessed through:

- Bayley Scales of Infant and Toddler Development (Bayley-III-NL) Cognition scale at 30 months [32].
- Wechsler Preschool and Primary Scale of Intelligence, Third and Fourth Edition (WPPSI-III-NL and WPPSI-IV-NL) at 5 years [33].
- Wechsler Intelligence Scale for Children, Third Edition (WISC-III-NL) at 8 and 12 years [34].

#### Attention

- Auditory attention at 5 years was tested through the Neuropsychological Assessment II (NEPSY-II) [35].
- Sustained attention at ages of 8 and 12 years was assessed by the Dot Cancellation Test (DCT) [36]. Both execution speed and fluctuations in execution speed are scored.
- Selective attention and cognitive flexibility at the ages of 8 and 12 years were assessed through the Stroop Interference Color Word Test [37].

#### Memory

- Short term auditory and visual memory at 5 years were assessed through the KAUFMAN Number Recall and Hand Movements Test [38].
- Verbal memory at 8 and 12 years was assessed through the 15 Words Test (15WT) [39].
- Visuospatial memory at 8 and 12 years was assessed through the Rey Complex Figure Test (RCFT). Immediate and delayed recall were scored [40].

#### Visuospatial processing

- Visuospatial processing at 8 and 12 years was assessed through RCFT Copy [40].

#### Executive functioning

- Planning and executive functioning skills at 8 and 12 years were assessed through:
  - BADS Key Search [41].
  - BADS Modified Six Elements [42].

### Motor function assessments

All motor function tests were administered by certified pediatric physical therapists, using the following motor performance assessment tools (detailed information in Supplemental file 1):

- Bayley Scales of Infant and Toddler Development (Bayley-III-NL) at 30 months tested the fine and gross motor scale, yielding a total (composite) score [32, 43].
- Movement Assessment Battery for Children (M-ABC 2-NL) was used at 5, 8 and 12 years [44]. The results of three subtests – manual dexterity, ball skills, and balance – yield a total motor function score.

### Statistical analysis

Data are presented as mean ± standard deviation (SD) for parametric data and median (interquartile range) for non-parametric data. Normal distribution of continuous variables was assessed using the Shapiro-Wilk test.

Neurodevelopment test scores – both cognitive and motor – were converted into z-scores where possible (general population: mean z-score = 0; standard deviation (SD) = 1); higher scores represent better performance.

#### Neurocognitive assessments

Data were analyzed using one-sample t-tests for intelligence index scores and IQ scores, comparing scores of participants with a norm mean of 100. For the neurocognitive tests in the domains of attention, memory, visuospatial processing, and executive functioning, mean z-scores were compared to a norm mean of 0 through the one-sample t-test. We assumed a 5% significance level.

#### Motor function assessments

Bayley-III fine, gross, and total motor scores were converted into z-scores and compared with a norm mean using the one-sample t-test. To analyze M-ABC 2 outcomes, raw scores were converted into z-scores. Manual dexterity, ball skill, balance, and total z-scores were compared with a norm mean using the one-sample t-test. Additionally, we calculated M-ABC 2 total motor percentile scores. Since a score below the 6^th^ percentile indicates a definite motor problem, we classified children as having a ‘definite motor problem’ when percentile scores were below 6 and as ‘no definite motor problem’ when percentile scores were 6 or higher. A ‘one-sample binominal test for proportion’ served to assess if ‘definite motor problems' occurred more often than expected (> 5%).

Missing data were handled using the pairwise deletion method. SPSS Statistics (version 25, IBM SPSS, Chicago, IL, USA) was used for data analysis.

## Results

### Patient characteristics

An overview of patient characteristics can be found in Table 1. In the study period, sixty-eight children underwent surgical CLA resection, of whom one died within the first week of life. Thus, 67 children were eligible for inclusion (Fig. 1). Three were excluded because CLA resection was performed secondarily to surgery for another major anatomical congenital malformation, for example correction of a congenital diaphragmatic hernia. Seventeen were excluded because follow-up data (at 30 months, 5, 8 or 12 years) were not available at any of the time points. Among the 47 included patients, 67 assessments were carried out, as 20 of these children were assessed at 2 time points (Fig. 1). Eight (18%) patients were prematurely born, and 7 (16%) patients were born with a low weight for gestational age. CPAM (N = 20, 43%) was the most common type of CLA, followed by BPS (N = 10, 21%), BC (N = 7, 15%) and CLO (N = 5, 11%). Two patients (4.3%) underwent surgery for ‘other’ lung abnormalities: one patient received an intra-thoracic implant because of lung agenesis and one patient underwent resection of a persisting pneumatocele. In three patients (6.4%), a combination of the above-mentioned abnormalities formed hybrid lesions.

**Table 1:** Patient characteristics

		**Patients** **N = 47**	**Assessments** **at 30 months** **N = 12**	**Assessments** **at 5 years** **N = 21**	**Assessments** **at 8 years** **N = 23**	**Assessments** **at 12 years** **N = 11**
Sex	Female	21 (45%)	8 (67%)	7 (33%)	8 (35%)	4 (36%)
	Male	26 (55%)	4 (33%)	14 (67%)	15 (65%)	7 (64%)
Gestational age (wk)		38.6 (± 1.8)	38.6 (± 1.5)	38.6 (± 1.8)	39.0 (± 1.7)	38.4 (± 2.0)
Prematurity^b^		8/44 (18%)	2/12 (17%)	3/19 (16%)	2/21 (9.5%)	2/10 (20%)
Birth weight (g)		3203 (± 531)	3143 (± 80)	3211 (± 136)	3337 (± 113)	3206 (± 194)
Low birth weight^c^		7/43 (16%)	2/12 (17%)	3/19 (16%)	2/20 (10%)	2/10 (20%)
Type CLA						
	CPAM	20 (43%)	4 (33%)	9 (43%)	9 (39%)	7 (64%)
	BPS	10 (21%)	1 (8.3%)	5 (24%)	6 (26%)	1 (9.1%)
	BC	7 (15%)	2 (17%)	4 (19%)	4 (17%)	1 (9.1%)
	CLO	5 (11%)	1 (8.3%)	3 (14%)	3 (13%)	1 (9.1%)
	Lung agenesis	1 (2.1%)	1 (8.3%)	0	0	0
	Other	1 (2.1%)	1 (8.3%)	0	0	0
	Combination^a^	3 (6.4%)	2 (17%)	0	1 (4.3%)	1 (9.1%)
Associated morbidity^d^						
	No	40 (85%)	9 (75%)	19 (91%)	20 (87%)	11 (100%)
	Yes	7 (15%)	3 (25%)	2 (9.5%)	3 (13%)	0
Symptom onset						
	Neonatal period (< 28d)	26 (55%)	7 (58%)	10 (48%)	11 (48%)	8 (73%)
	After neonatal period (> 28d)	21 (45%)	5 (42%)	11 (52%)	12 (52%)	3 (27%)
Age at surgery						
	< 12 months	34 (74%)	8 (73%)	17 (81%)	17 (74%)	8 (73%)
	> 12 months	12 (26%)	3 (27%)	4 (19%)	6 (26%)	3 (27%)
	Unknown	1	1	0	0	0
Surgical approach						
	Thoracotomy	27 (57%)	8 (67%)	10 (48%)	10 (43%)	10 (91%)
	Thoracoscopy	20 (43%)	4 (33%)	11 (52%)	13 (57%)	1 (9.1%)
	Unknown	0	0	0	0	0

Data presented as N (%), median (interquartile range) or mean (± SD)

Of 20 included patients, clinical characteristics were used for analysis at different assessment ages

*CPAM* Congenital Pulmonary Airway Malformation, *BPS* Bronchopulmonary Sequestration, *BC* Bronchogenic Cyst, *CLO* Congenital Lobar Overinflation

^a^Combination (N = 3): Lung agenesis + BPS (N = 1), CPAM + BPS (N = 1), CPAM + CLO (N = 1)

^b^Prematurity: gestational age < 37 weeks

^c^Low birth weight: birth weight < P10 for gestation

^d^Associated morbidities: cardiac (N = 3: atrium septum defect, aortic arch deformity), neurologic (N = 2: cerebral bleeding, filamin A mutation), syndromic (N = 2: Klinefelter syndrome, 47 XXX) or other (N = 1: ovarian cysts, hip dysplasia) origin

Non CLA-related associated morbidities were present in 7 (15%) children. CLA-related symptoms occurred in the neonatal period in 26 (55%) children. Manifestations of CLA included respiratory insufficiency (N = 24), recurrent respiratory tract infections (N = 8), mediastinal shift (N = 4), cardiac overload (N = 3), and feeding problems (N = 2). Most of the resections were carried out in the first year of life (74%). The mean age at surgery of the 12 children who underwent resection later in life was 2.5 years. Overall, thoracotomy (57%) was the most frequent surgical approach*.*

### Neurodevelopmental outcomes

Across all ages and neurodevelopmental tests, there was a 8.6% rate of missing data. This was partially due to patient fatigue and lack of cooperation during the tests. Other causes included the cognitive or physical inability to complete the tests and missing a follow-up appointment.

### Neurocognitive outcomes

#### Intelligence

On group level, intelligence was not significantly impaired compared to the general population, as mean total IQ scores at 30 months, 5, 8 and 12 years did not significantly differ from norm values (Table 2).

**Table 2:** Neurocognitive outcomes^b^

*Intelligence*			Mean intelligence (± SD)	95% CI of difference	IQ < 85^a^	IQ < 70^a^
	30 months (N = 10)		93 (± 12)	[-15.4; + 2.0], p = 0.12	2 (20%)	1 (10%)
	5 years (N = 20)		101 (± 14)	[-5.2; + 8.0], p = 0.66	3 (15%)	0
	8 years (N = 22)		107 (± 19)	[-1.5; + 15.0], p = 0.11	1 (4.5%)	1 (4.5%)
	12 years (N = 11)		102 (± 20)	[-11.8; + 15.4], p = 0.77	2 (18%)	1 (9.1%)

*Attention*			Mean z-scores	95% CI of difference		
	8 years					
		DCT speed (N = 21)	**-2.4**	**[-4.1; -0.8], p = 0.006**		
		DCT fluctuations (N = 21)	**-7.1**	**[-12.8; -1.4], p = 0.02**		
		Stroop interference (N = 18)	-0.3	[-0.9; 0.2], p = 0.21		
	12 years					
		DCT speed (N = 11)	-2.1	[-4.7; 0.4], p = 0.09		
		DCT fluctuations (N = 11)	-0.7	[-2.3; 0.9], p = 0.33		
		Stroop interference (N = 11)	-0.4	[-1.0; 0.3], p = 0.22		

*Memory*			Mean z-scores	95% CI of difference		
	5 years					
		Kaufman auditory (N = 19)	0.4	[-0.02; 0.7], p = 0.06		
		Kaufman visual (N = 19)	0.7	[0.3; 1.0], p < 0.001		
	8 years					
		15 WT immediate (N = 21)	0.2	[-0.2; 0.7], p = 0.32		
		15 WT recall (N = 21)	-0.3	[-0.8; 0.2], p = 0.21		
		RCFT recall (N = 20)	**-1.0**	**[-1.5; -0.5], p < 0.001**		
	12 years					
		15 WT immediate (N = 11)	0.5	[-0.3; 1.4], p = 0.21		
		15 WT recall (N = 11)	0.2	[-0.4; 0.9], p = 0.45		
		RCFT recall (N = 11)	-0.2	[-0.8; 0.4], p = 0.53		

*Visuospatial processing*			Mean z-scores	95% CI of difference		
	8 years					
		RCFT copy (N = 20)	0.0	[-0.5; 0.4], p = 0.92		
	12 years					
		RCFT copy (N = 11)	0.2	[-0.1; 0.6], p = 0.19		

*Executive functioning*			Mean z-scores	95% CI of difference		
	8 years					
		BADS Key Search (N = 20)	0.1	[-0.3; 0.6], p = 0.54		
		BADS Modified Six Elements (N = 15)	**-0.4**	**[-0.7; -0.1], p = 0.01**		
	12 years					
		BADS Key Search (N = 11)	0.9	[0.1; 1.7], p = 0.04		
		BADS Modified Six Elements (N = 8)	**-1.0**	**[-1.5; -0.4], p = 0.006**		

*p* < 0.05 is considered statistically significant. Values in bold indicate significantly worse than expected performance in the assessed group

*N* Number of patients,* SD* Standard deviation,* DCT* Dot Cancellation Test, *RCFT* Rey Complex Figure Test, *15 WT* 15 Word Test

^a^An IQ score of < 85 marks a deviation of < -1 standard deviation (SD), an IQ score of < 70 equals < -2 SD

^b^Neurocognitive performance of patients is measured against the general population. Scores of patients are compared with norm values that represent the mean in the general population. For the general population, the expected mean intelligence index score is 100 and expected mean z-scores are 0. Calculated differences between patients and the general population and associated 95% Confidence Intervals (CI) are presented

#### Attention

At 5 years, the auditory attention test from the NEPSY battery revealed no significant impairments. All 18 assessed children had percentile scores within the ‘average’ range or above.

For sustained attention, at 8 years, mean DCT z-scores for execution speed and fluctuations were significantly different compared to the norm values, respectively -2.41; [-4.05; -0.77], p = 0.006 and -7.11; [-12.79; -1.43], p = 0.02, indicating sustained attention impairments at this age (Table 2).

At 12 years, mean DCT z-scores were not significantly below norm values. At 8 and 12 years, mean Stroop selective attention z-scores were not significantly impaired. Three children underwent sustained attention assessment at both 8 and 12 years; only one of them showed persistent impairment (Table 2).

#### Memory

At the age of 5 years, none of the children showed auditory memory impairment; overall, they scored significantly above average on the visual short term memory tests (mean z-score 0.7; [0.3; 1.0], p < 0.001) (Table 2).

At 8 and 12 years, mean z-scores of verbal memory (15 WT) did not significantly differ from the norm values (Table 2). At 8 years, the mean RCFT recall z-score was -1.0 ([-1.5; -0.5], p < 0.001). At 12 years, the mean RCFT recall score was no longer below average (Table 2).

#### Visuospatial processing

At 8 and 12 years, the mean RCFT copy z-scores for visuospatial processing were not significantly impaired (Table 2).

#### Executive functioning

At 8 and 12 years, on group level, the results of the BADS Key Search test were not impaired, thus not consistent with visuospatial planning defects (Table 2). At both 8 and 12 years, scores on the BADS Modified Six Elements tests were below average (p value of 0.01 and 0.006 respectively) (Table 2).

### Motor function outcomes

On a group level, significant impairments of motor performance were not noted across the assessed ages. The mean z-scores of Bayley-III-NL’s fine, gross, and total motor scale, as well as M-ABC 2’s manual dexterity, ball skill, balance, and total motor scale did not significantly differ from the norm population mean at any of the tested ages (Table 3)*.* At ages 5 and 12 years, scores of respectively 5.0% and 0% of the children were consistent with definite motor problems, similar to the expected proportions. However, four of the 8-year-olds (18%) had a definite motor problem, which proportion was significantly higher than the expected 5% (95% CI of proportion [0.052; 0.403], p = 0.022).

**Table 3:** Motor function outcomes^a^

	N	Mean z-scores	SD	95% CI	p-value
**30 months**					
Total motor score	10	-0.3	1.2	[-1.1; 0.6]	0.46
Fine motor score	9	-0.3	1.1	[-1.2; 0.5]	0.38
Gross motor score	9	-0.5	1.3	[-1.4; 0.5]	0.28
**5 years**					
Total motor score	20	0.2	1.0	[-0.3; 0.7]	0.34
Manual dexterity	19	0.2	0.7	[-0.1; 0.5]	0.18
Ball skills	19	-0.1	0.8	[-0.4; 0.3]	0.70
Balance	19	0.2	1.1	[-0.4; 0.7]	0.50
**8 years**					
Total motor score	21	0.1	1.2	[-0.4; 0.7]	0.62
Manual dexterity	22	0.2	1.1	[-0.3; 0.7]	0.52
Ball skills	22	0.0	0.7	[-0.3; 0.4]	0.85
Balance	21	0.1	1.4	[-0.6; 0.7]	0.79
**12 years**					
Total motor score	11	0.1	0.7	[-0.4; 0.6]	0.59
Manual dexterity	11	0.4	0.9	[-0.3; 1.0]	0.23
Ball skills	11	-0.1	0.6	[-0.5; 0.3]	0.55
Balance	11	-0.2	0.8	[-0.7; 0.3]	0.38

At the ages of 30 months and 5 years, the total motor performance score of one patient (N = 1) was documented in the electronic patient files but scores of the subtests were not available due to administrative reasons

*p* < 0.05 is considered statistically significant

*N* Number of patients,* SD* Standard deviation

^a^Motor performance of patients is measured against the general population. Scores of patients are compared with norm values that represent the mean in the general population. For the general population, the expected mean z-scores are 0. Calculated differences between patients and the general population and associated 95% Confidence Intervals (CI) are presented

Evaluation of motor subskills revealed that all four children with a definite motor problem at 8 years had balance problems, and that two also had manual dexterity problems whereas none of them had impaired ball skills. Three out of those four children had also been evaluated at 5 years; only one of them showed definite motor problems at that age. None of the four patients with a definite motor problem at 8 years were examined at 12 years.

## Discussion

To the best of our knowledge, this is the first analysis of neurocognitive and motor performance in children who underwent resection of CLA early in life. We analyzed neurocognitive and motor performance of participants of a prospective longitudinal follow-up program at the ages of 30 months, 5, 8, and 12 years. Intelligence testing and visuospatial processing results did not significantly differ from Dutch reference values at any of the tested ages. At the ages of 8 and 12 years, memory and executive functioning subtests revealed some below-average scores, but the results were not indicative of general impairments, as effect sizes were relatively small and other subtests were not impaired. Next to this, significant impairments in sustained attention and visuospatial memory were noted at 8 years, though not in all the tests. Interestingly, these mean z-scores were normal at 12 years. Three children underwent sustained attention assessment at both 8 and 12 years; only one of them showed persistent impairment. The motor performance results were generally normal. Nevertheless, more 8-year-olds than expected had total scores indicative of definite motor problems; mainly balance and manual dexterity were affected. None of these 8-year-olds were also assessed at 12 years of age; thus, longitudinal data on the persistence of motor performance problems were not available.

Our data may help improve the counselling of patients and their caregivers on the expected long-term neurodevelopment following surgery for CLA. Moreover, based upon our results we see limited value of routine extensive neurodevelopmental assessments throughout childhood in children that have undergone resection of a CLA lesion. However, we do recommend enrolling all children with CLA – resected and non-resected – in a structured long-term follow-up program with intervals of several years in between visits, with evaluation of growth, general wellbeing, imaging, exercise capacity and pulmonary function. In addition, we suggest at least considering neurodevelopmental assessment in case of apparent additional morbidities at birth or a history of neonatal critical illness. Similarly, neurodevelopmental tests should be performed if parents or teachers are concerned about the neurocognitive or motor functioning of a child with CLA. The importance of detecting possible neurodevelopmental problems in an early stage lies in the chance to prevent worsening of existing impairments by being able to offer these patients additional medical or paramedical care such as physiotherapy.

When we examined more profoundly the medical history of the four children with definite motor problems at 8 years, we found several possible extra pulmonary grounds of impaired motor scores. First, two of them had associated congenital anomalies and prolonged hospitalization during the first month of life that could possibly contribute to the disturbed motor performance: Filamin A deficiency [45, 46] and Klinefelter syndrome [47]. Second, three of them showed impaired sustained attention, possibly indirectly influencing motor scores. Third, one of them did not participate in the sustained and selective attention assessments because the reading and counting skills were insufficient to adequately perform these tests. As this child’s IQ was 68, this deficit in reading and counting may well be associated with his/her cognitive impairment, which in turn could have influenced motor function assessment.

Several important potential drawbacks associated with this study need to be addressed. First, the sample size was relatively small, especially when distributed over the different ages at which assessment took place. This drawback is frequently encountered in follow-up studies of rare congenital anomalies, limiting the certainty with which the results can be interpreted and generalized. However, for two decades we have been enrolling patients with major congenital anomalies in a structured prospective surgical longitudinal follow-up program that is being offered as part of the standard of care at our tertiary university hospital, leading to this relatively large cohort [31]. Furthermore, we were unable to perform sub analyses for possible confounders such as gestational age, birth weight, surgical approach, CLA type, or associated morbidities, since results of the several neurodevelopmental tests used across the different ages were often not interchangeable and consequently could not be uniformly analyzed. The combination of the abovementioned small sample size and variation in neurodevelopmental testing prevented us from performing longitudinal analyses of those children that underwent tests at consecutive ages. Theoretically we could have performed a longitudinal analysis of motor performance, since all children were tested through the Movement-ABC 2. However, only 13 children were tested at 5 and 8 years, only 3 were tested at 8 and 12 years, and not one was tested at all three consecutive ages. Therefore, longitudinal analysis was considered futile. For the improvement of practical feasibility of longitudinal analyses in the future, we suggest implementation of uniform tests with interchangeable results. An additional limitation was the management of missing data through the pairwise deletion method, in which cases are excluded from a single analysis if no data is available. We are aware that pairwise deletion is susceptible to potential bias. However, using the pairwise deletion method we wanted to maximize the use of all available data for each specific analysis, as our sample size was limited. Finally, considering the ongoing discussion of whether or not to operate asymptomatic CLA – we could not compare the neurodevelopmental outcomes between surgically and conservatively managed cases, as the latter group is enrolled in a less extensive follow-up program without routine neurodevelopmental assessment. As a result, we could not determine the extent to which surgery itself may contribute to the development of neurodevelopmental impairments.

In conclusion, this evaluation reveals generally normal neurodevelopmental outcomes for children who have undergone resection of CLA compared to their healthy age peers. Hence, we suggest a structured follow-up program throughout childhood and beyond, which evaluates growth, general wellbeing, imaging, exercise capacity and pulmonary function. Neurodevelopmental assessment should only be considered when additional risk factors are present, for example other major congenital malformations or a complicated neonatal course, or if parents or teachers are concerned about impaired development within these domains, hereby introducing a tailor-made approach.

### Supplementary Information

Below is the link to the electronic supplementary material.

Supplementary file1 (PDF 574 KB)

## Data Availability

Source data are stored on a secure internal drive and are available upon request.
