# Supplementary material for: Long-term neurodevelopment in children with resected congenital lung abnormalities
Source: Eur J Pediatr. 2023 Jun 16;182(9):3845–55. doi: 10.1007/s00431-023-05054-5 (PMC10570195; doi:10.1007/s00431-023-05054-5)
Supplement: Supplementary file 1 — Supplementary file1 (PDF 574 KB) [file 431_2023_5054_MOESM1_ESM.pdf]

Supplemental file 1: Description of the neurodevelopmental tests used

Intelligence

The deployed intelligence tests yield a total intelligence index score, with a normalized population mean of 100 and a standard deviation of 15.

Bayley Scales of Infant and Toddler Development (Bayley-III-NL)
The cognition scale of the third edition Bayley Scales of Infant and Toddler Development (Bayley-III-NL) was used at 30 months of age to test patients' cognitive development (1).

Wechsler Preschool and Primary Scale of Intelligence, Third and Fourth Edition (WPPSI-III and -IV)
The Wechsler Preschool and Primary Scale of Intelligence, Third and Fourth Edition (WPPSI-III and -IV) were used at 5 years of age, assessing intellectual functioning on different domains, including verbal comprehension, perceptual organization, and processing speed (2).

Wechsler Intelligence Scale for Children (WISC-III-NL)
The Dutch version of the Wechsler Intelligence Scale for Children, Third Edition (WISC-III-NL) was administered at 8 and 12 years of age, testing intelligence (3). The total IQ score is based on subtests of the domains verbal comprehension, perceptual organization, and processing speed.

Attention

NEPSY Auditory Attention
The auditory attention test from the NEPSY battery was administered to test attention at the age of 5 years (4). The test subject listens to a prerecorded auditory stimulus of a list of words and is instructed to touch the appropriate circle in the stimulus book when he or she hears a target word.

The Dot Cancellation Test (Bourdon-Vos)
This test, suited for children aged 6 to 17 years – was used to measure sustained attention at ages of 8 and 12 years (5). In this test, the examinee is instructed to cross off all figures with four dots on a sheet with 33 rows of figures, as precisely and as fast as they can. Speed and fluctuations in speed are scored.

Stroop Interference Color Word Test
The Stroop Interference Color Word Test served to measure selective attention and cognitive flexibility at the ages of 8 and 12 years. The assessment consists of three trials: in the first trial the subject must read color names, in the second trial he/she names printed colors, and in the third trial the examinee is instructed to name printed colors not denoted by the color name (6).

Visuospatial processing

Rey Complex Figure Test (RCFT) Copy
The Rey Complex Figure Test (RCFT) was used to evaluate visuospatial constructional processing at 8 and 12 years. Examinees are shown a complex figure and are asked to reproduce this figure as accurately as possible (9).

Memory

Kaufman Assessment Battery for Children (K-ABC): short term auditory and visual memory
Two Kaufman subtests were used to assess short term memory at 5 years (7). It measures two domains of short term memory:

- Subtest number recall (Auditory): repeating a series of numbers (2 to 9) read aloud by the assessor.
- Subtest hand movements (Visual): copying a series of hand gestures shown by the assessor.

15 Words Test (Dutch version of Rey Auditory Verbal Learning Test)
The 15 Words Test (15WT) was used to investigate verbal memory problems at the ages of 8 and 12 years. Fifteen words have to be memorized during five trials. After twenty minutes, the participant is asked to name as many words as they can remember (8).

Rey Complex Figure Test (RCFT) Recall
The Rey Complex Figure Test (RCFT) also served to evaluate visuospatial memory at 8 and 12 years. Test subjects are instructed to draw a figure from memory after a delay of 30 minutes (9).

Executive functioning

BADS Key Search test
The BADS key search test was administered to test executive functioning skills at the ages of 8 and 12 years (10). The test subject is asked to demonstrate how they would search a field for a set of lost keys and their strategy is scored according to its functionality.
BADS Modified Six Elements test
The test-subject is asked to work on six different tasks for which they have five minutes. The child needs to make sure that by the end of the five minutes, all six of the tasks have been completed as far as possible. This is a test of planning, task scheduling and performance monitoring (11).

Motor performance

Bayley Scales of Infant and Toddler Development (Bayley-III-NL)
The Bayley-III-NL motor scale tests fine and gross motor skills (1, 12).

- Fine motor skills: reaching, grabbing, visual-motor integration, motor planning, speed.
- Gross motor skills: static positioning, movements of trunk and limbs, balance.

The Bayley-III-NL motor scale yields a total motor performance score based on the abovementioned fine and gross motor scores, with a normalized population mean of 100 and a standard deviation of 15.

Movement Assessment Battery for Children (M-ABC 2-NL)
The Dutch second version of the Movement Assessment Battery for Children (M-ABC 2-NL) was used to assess motor function skills at 5, 8, and 12 years. This assessment tool has been validated for use in children of 3-16 years old and yields motor function scores that can be converted into percentile scores. The total motor function percentile score is based on the percentile results of three subtests:

manual dexterity (representing fine motor skills), aiming & catching and balance (the latter two representing gross motor skills). A total motor function score ranging from 100th-16th percentile is considered ‘normal’, a score between the 15th-6th percentile is considered a ‘borderline motor problem’, and everything below the 5th percentile is defined as ‘definite motor problem’ (13).

Validated Dutch versions of all abovementioned tests were used

References

1. Bayley N. Manual for the Bayley Scales of Infant Development. In: Corporation P, editor. 1969.
2. Wechsler D. Wechsler Preschool and Primary Scale of Intelligence-Fourth Edition (WPPSI-IV): Technical and Interpretive Manual. In: NCS Pearson I, editor. Bloomington, MN2012.
3. Wechsler D. Wisc-iii handleiding. The Psychological Corporation, London. 2002.
4. Korkman MK, U.; Kemp, S. NEPSY-II-NL. Test om de neuropsychologische ontwikkeling van kinderen van 5 t/m 12 jaar in kaart te brengen. In: Clinical P, editor. 2010.
5. Vos P. Bourdon-Vos. Handleiding (Manual Dot Cancellation Test). Lisse, Swets en Zeitlinger; 1992.
6. Schmand B. HP, De Koning I. Normen van psychologische tests voor gebruik in de klinische neuropsychologie. In: Psychologen NIv, editor. 2012.
7. Kaufman ASK, N.L. Kaufman Assessment Battery for Children. 1983.
8. Kok A. Episodic memory. In: UMC A, editor. 2019.
9. Shin MS, Park SY, Park SR, Seol SH, Kwon JS. Clinical and empirical applications of the Rey-Osterrieth Complex Figure Test. Nat Protoc. 2006;1(2):892-9.
10. Wilson. Behavioural assessment of dysexecutive syndrome, BADS. 1996.
11. Baron IS. Behavioural Assessment of the Dysexecutive Syndrome for Children (BADS-C) by Emslie, H., Wilson, F. C., Burden, V., Nimmo-Smith, I., & Wilson, B. A. (2003). Child Neuropsychology. 2007;13(6):539-42.
12. van Baar ALS, L.J.P.; Verhoeven, M. Bayley-III-NL | Motoriek Schaal. In: Utrecht U, editor.: Pearson Clinical.
13. Henderson SES, D.A. The Movement Assessment Battery for Children: Manual. In: Corporation TP, editor. San Antonio, TX1992.
